# Draft Genome Sequence of *Thalassospira xiamenensis* Strain MCCC 1A03042^T^

**DOI:** 10.1128/genomeA.01702-16

**Published:** 2017-03-02

**Authors:** Meiqing Li, Shuo Yang, Qiliang Lai, Zongze Shao

**Affiliations:** State Key Laboratory Breeding Base of Marine Genetic Resources, Key Laboratory of Marine Genetic Resources, Third Institute of State Oceanic Administration, Collaborative Innovation Center for Development and Utilization of Marine Biological Resources, Key Laboratory of Marine Genetic Resources of Fujian Province, Xiamen, China

## Abstract

*Thalassospira xiamenensis* strain MCCC 1A03042^T^ was isolated from deep-sea sediment of the Indian Ocean, and it was characterized with heavy-metal arsenic tolerance. Here, we present the draft genome of strain MCCC 1A03042^T^, which contains 4,786,207 bp with a G+C content of 52.6% and 4,359 protein-coding genes.

## GENOME ANNOUNCEMENT

The species *Thalassospira xiamenensis* was first proposed by Liu et al. (1). Members of the *T. xiamenensis* species are non-spore-forming, Gram-negative, curved rods that are motile by means of one polar ﬂagellum. *T. xiamenensis* strain MCCC 1A03042^T^ is closely related to *T. xiamenensis* strain M-5^T^, which was the type strain, and the similarity was 99.716%. Strain MCCC 1A03042^T^ was isolated from deep-sea sediment of the Indian Ocean, plated directly on 2216E agar (2) and incubated at 22°C. We found that it was characterized with heavy-metal arsenic tolerance and grew in the medium with an arsenic concentration of 10 mM.

Genomic DNA was puriﬁed from *T. xiamenensis* MCCC 1A03042^T^ with an AxyPrep bacterial genomic DNA miniprep kit (Axygen) according to the manufacturer’s instructions. The genome sequence was determined by Shanghai Majorbio Bio-pharm Technology Co., Ltd. (Shanghai, China) using Solexa paired-end sequencing technology. A total of 500 Mb of clean data (500-bp library) were generated to reach about 100-fold depth of coverage with an Illumina/Solexa Genome Analyzer IIx (Illumina, San Diego, CA, USA). The genome of *T. xiamenensis* MCCC 1A03042^T^ consists of 72 contigs of 4,786,207 bp with an average G+C content of 52.6%.

Gene prediction and annotation of the draft genome were carried out using the NCBI Prokaryotic Genome Annotation Pipeline (PGAP) (http://www.ncbi.nlm.nih.gov/genomes/static/Pipeline.html) (3). In total, 4,470 genes, 4,359 of which are protein-coding genes, and 50 tRNAs were predicted; 59 pseudogenes were also identiﬁed. Genome annotation and analysis were also done using the RAST server (http://rast.nmpdr.org) (4). Four genes (*ars*) for arsenate reduction were found in the annotations. A previous study demonstrated that an initial step in arsenic metabolism is the reduction of arsenate (5). In addition, the presence of an arsenic transporter was also noticed.

### Accession number(s).

The draft genome sequence of *T. xiamenensis* MCCC 1A03042^T^ has been deposited at GenBank under the accession number LPXM00000000. The version described in this paper is the ﬁrst version, LPXM01000000.
